# Supplementary material for: Inflammatory factors and risk of lung adenocarcinoma: a Mendelian randomization study mediated by blood metabolites
Source: Front Endocrinol (Lausanne). 2024 Aug 27;15:1446863. doi: 10.3389/fendo.2024.1446863 (PMC11384989; doi:10.3389/fendo.2024.1446863)
Supplement: Supplementary file 1 [file DataSheet1.zip › supplementary files/S2/GCST90201012/sensitivity-analysis.pdf]

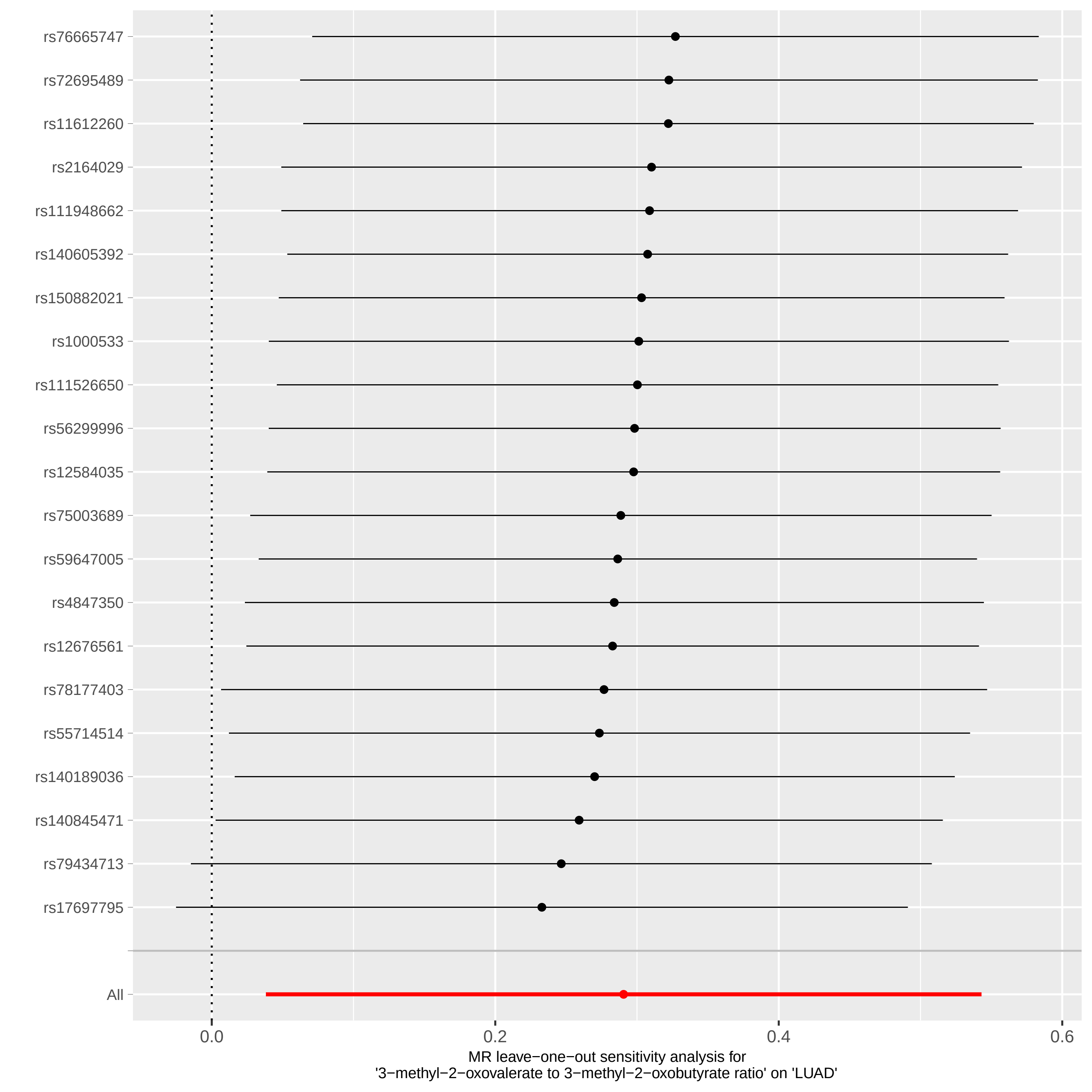
rs76665747
rs72695489
rs11612260
rs2164029
rs111948662
rs140605392
rs150882021
rs1000533
rs111526650
rs56299996
rs12584035
rs75003689
rs59647005
rs4847350
rs12676561
rs78177403
rs55714514
rs140189036
rs140845471
rs79434713
rs17697795
All
0.0
0.2
0.4
0.6
MR leave−one−out sensitivity analysis for
'3−methyl−2−oxovalerate to 3−methyl−2−oxobutyrate ratio' on 'LUAD'
